# Cerebellar Volumes Associate with Behavioral Phenotypes in Prader-Willi Syndrome

**DOI:** 10.1007/s12311-020-01163-1

**Published:** 2020-07-13

**Authors:** Kenichi Yamada, Masaki Watanabe, Kiyotaka Suzuki, Yuji Suzuki

**Affiliations:** Center for Integrated Human Brain Science, Brain Research Institute, University of Niigata, 1-757, Asahimachi, Chuo-ku, Niigata 9518585 Japan

**Keywords:** Autism, Cerebellum, Dentate nucleus, Hyperphagia, Obesity, Prader-Willi syndrome

## Abstract

The objective of this study was to investigate lobule-specific cerebellar structural alterations relevant to clinical behavioral characteristics of Prader-Willi syndrome (PWS). We performed a case-control study of 21 Japanese individuals with PWS (age; median 21.0, range 13–50 years, 14 males, 7 females) and 40 age- and sex-matched healthy controls with typical development. Participants underwent 3-Tesla magnetic resonance imaging. Three-dimensional T_1_-weighted images were assessed for cerebellar lobular volume and adjusted for total intracerebellar volume (TIV) using a spatially unbiased atlas template to give a relative volume ratio. A region of interest analysis included the deep cerebellar nuclei. A correlation analysis was performed between the volumetric data and the clinical behavioral scores derived from the standard questionnaires (hyperphagia, autism, obsession, and maladaptive index) for global intelligence assessment in paired subgroups. In individuals with PWS, TIV was significantly reduced compared with that of controls (*p* < 0.05, family-wise error corrected; mean [standard deviation], 1014.1 [93.0] mm^3^). Decreased relative lobular volume ratios were observed in posterior inferior lobules with age, sex, and TIV as covariates (Crus I, Crus II, lobules VIIb, VIIIa, VIIIb, and IX). However, increased ratios were found in the dentate nuclei bilaterally in individuals with PWS (*p* < 0.01); the mean (standard deviation) × 10^−3^ was as follows: left, 1.58 (0.26); right, 1.67 (0.30). The altered lobular volume ratios showed negative correlations with hyperphagic and autistic characteristics and positive correlations with obsessive and intellectual characteristics. This study provides the first objective evidence of topographic patterns of volume differences in cerebellar structures consistent with clinical behavioral characteristics in individuals with PWS and strongly suggests a cerebellar contribution to altered functional brain connectivity in PWS.

**Electronic supplementary material:**

The online version of this article (10.1007/s12311-020-01163-1) contains supplementary material, which is available to authorized users.

## Introduction

Prader-Willi syndrome (PWS) is a complex multisystem genetic disorder that is characterized by a specific developmental trajectory, which includes hypotonia, developmental delay, hyperphagia typically causing obesity, and a higher vulnerability to maladaptive behavior [1–4]. These symptoms typically develop in early life in a phase-dependent manner and frequently overlap with other features such as autism spectrum disorder (ASD), obsessive compulsive disorder, and depression, all of which have been shown to be more pronounced toward adulthood [5–7]. Therefore, these diverse characteristics strongly suggest that the behavioral characteristics are based not only on merely functional developmental alterations but also on structural brain developmental alterations starting early in life.

Previous magnetic resonance imaging (MRI) studies have noninvasively detected in vivo structural brain alterations in individuals with PWS. Morphometric analysis has demonstrated developmental abnormalities in cortical and subcortical brain structures on a macroscopic scale [8, 9], and these findings have been supported by microstructural evidence of altered connectivity using diffusion tensor imaging [10, 11]. In contrast, research on the contribution of the cerebellum in PWS remains sparse, despite evidence suggesting a significant role of the cerebellum in a wide variety of neurodevelopmental disorders [12]. While reduced cerebellar volumes have been observed in PWS [13], more finely detailed lobular-scale structural analyses are required for a better understanding of the clinical behavioral patterns in PWS. Given the cerebellum having multiple functional domains, those specific domains can therefore be related to the various behavioral patterns observed in PWS.

The cerebellum occupies a role in non-motor functions (e.g., executive function, linguistic processing, spatial cognition, and affect regulation), and deficits in these functions contribute to cerebellar cognitive affective syndrome (CCAS), also known as Schmahmann’s syndrome [14]. The topography of CCAS has been attributed to damage to the posterior lobules, which may occur together with motor or vestibular syndromes due to the distribution damage [15]. Recently, defining features of CCAS have been validated using neurocognitive assessment based on a wide range of patients with cerebellar deficits and cognitive impairment [16]. Accordingly, the utility of clinical assessment-based approaches encourages us to investigate the correlation between cerebellar lobules and nuclei structures and the aforementioned characteristic behaviors in PWS.

Recently, advanced morphometric imaging based on a probabilistic atlas, the spatially unbiased infratentorial template (SUIT), has been introduced [17–19]. This method enables the visualization of cerebellar lobule-dependent alterations associated with the different functional contributions of each cerebellar structure. These have been applied to elucidate cerebro-cerebellar correlates in a wide range of neurological disorders [20, 21]. Therefore, this advanced method could provide detailed information regarding the contribution of the cerebellum to PWS and potentially reveal correlations between clinical behavioral variables and altered connectivity in patients with PWS.

In this study, we utilized SUIT based on a 3-Tesla MRI system to detect cerebellar developmental abnormalities in individuals with PWS in a lobule-specific manner. We aimed to test the hypothesis that individuals with PWS have developmental abnormalities in cerebellar volume sufficient to produce altered functional connectivity.

## Materials and Methods

### Participants

In total, 21 individuals with PWS (age; median 21.0, range 13–50 years, 14 males, 7 females) and 40 age- and gender-matched control individuals with typical development (TD) participated in the study. All participants with PWS were recruited from support groups for PWS in Japan, together with the TD control individuals from a regional community around our university. The primary characteristics of participants are summarized in Table 1. Written informed consent and child assent were obtained from all participants and parents or guardians before any aspect of the research was initiated. The study was conducted according to the human research guidelines of the institutional review board of the University of Niigata under the approval of the Research Ethics Committee (approval number # 2482) and in accordance with the 1964 Helsinki declaration and its later amendments or comparable ethical standards.

**Table 1:** Characteristics of the participants

Sample	PWS	Control	*p*^a^
*n*	21	40	-
Age, year; median (range)	21.0 (13–50)	21.0 (9–48)	0.083
Sex, M/F	14/7	25/15	-
Handedness^b^	80.6 (44.1)	85.9 (21.1)	0.854
Body weight, *z* score	0.5 (1.8)	− 0.2 (0.8)	0.183
Genetic subtype, del/upd	19/2	-	-
Complications	T2DM 1	none	-
Medications in use at study time			
Growth hormone	RhGH 4	-	-
Antipsychotics	Aripiprazole 1	-	-
Paired subgroups			
*n*	12	13	-
Age, year; median (range)	24.5 (13–50)	25.0 (12–48)	0.498
Sex, M/F	8/4	7/6	-
Handedness^b^	76.3 (56.5)	89.6 (13.7)	0.875
Body mass index	29.0 (8.1)	20.1 (3.1)	< 0.001
Behavioral characteristics, total score			
Hyperphagia questionnaire	20.9 (5.7)	12.6 (2.7)	< 0.001
Autism spectrum quotient	23.9 (5.9)	16.4 (8.1)	0.038
Leyton obsessional inventory	15.6 (16.0)	32.8 (22.1)	0.047
Kohs block-test IQ	60.3 (17.4)	116.9 (9.6)	< 0.001
VABS, maladaptive score	19.3 (2.3)	13.6 (0.5)	< 0.001

^a^Mann-Whitney rank sum test, ^b^laterality quotient derived from the Edinburgh handedness inventory

Data are presented as *n* or mean (standard deviation) unless otherwise stated. Control, typical development; del, deletion; IQ, intelligence quotient; PWS, Prader-Willi syndrome; RhGH, recombinant human growth hormone; T2DM, type 2 diabetes mellitus; upd, uniparental disomy; VABS, Vineland adaptive behavior scale, second edition

The clinical diagnoses of PWS were confirmed by genetic testing according to clinical diagnostic criteria [2]. Semi-structured interviews were conducted for all individuals to determine past history, medical treatment, and behavioral characteristics. Handedness was assessed using the Edinburgh handedness inventory. Body weight was *z* score transformed using age- and sex-dependent average and standard deviation values derived from the national statistics database (National Health and Nutrition Survey. e-stat. Tokyo: 2018. Available from: https://www.e-stat.go.jp/). Other psychiatric disorders were identified according to *the Diagnostic and Statistical Manual of Mental Disorders*, fifth edition [22].

None of the participants had a history of other neurological diseases or of traumatic brain injury requiring surgical intervention. Control individuals with TD underwent neurological examination by interview to confirm neurologic normality and the absence of developmental abnormalities. Parents or guardians of TD controls were minimally interviewed and only contributed if further information pertaining to the subject’s early childhood was required. None of the participants consumed illicit drugs or alcohol.

The second part of the neuroimaging-behavior correlation study included 12 individuals with PWS and 13 TD controls. These participants were interviewed for behavioral characteristics observed during the developmental process since birth. Specific characteristics of these participants are summarized in the paired subgroup section of Table 1. Body mass index (BMI) was calculated from body height and weight measurements. Specific behavioral domains were assessed with the following: (1) the hyperphagia questionnaire (HQ) [23], Japanese version; (2) the autism-spectrum quotient (AQ) [24], Japanese version; and (3) the Leyton obsessional inventory (LOI) [25], Japanese version. Global intellectual functioning was assessed using the Kohs block design test to estimate the intelligence quotient (Kohs_IQ) [26]. Finally, the Vineland adaptive behavior scales (VABS; second Japanese edition) were administered [27], and the maladaptive score was extracted (VABS_mal) to assess global adaptive functioning under multidisciplinary follow-up support.

### Imaging Procedure

A horizontal 3-Tesla MRI system (General Electric Healthcare, Milwaukee, WI) with an 8-channel phased-array head coil was utilized for all imaging studies. The structural T_1_ images were acquired with an inversion recovery-prepared, three-dimensional spoiled-gradient echo sequence with the following parameters: axial slices, 20 × 20 mm; matrix, 512 × 512; slice thickness, 1.5 mm; interslice gap, 0 mm; echo time, 3.22 ms; repetition time, 7.744 ms; inversion time, 450 ms; flip angle, 20 degrees; and number of excitations, 1. The selected slices completely covered all regions from above the top of the head to the level of the foramen magnum. The average scan time was approximately 6 min.

For the imaging procedures with TD children or individuals with PWS, an original preparation protocol was applied using the “Zero-tesla” mock scanner preparation system, which we have developed in-house for children and individuals with neurodevelopmental disorders. Using audiovisual aids, the participants could watch their favorite movies or cartoons while lying inside the mock scanner to maximize comfort and effectively reduce anxiety in the real MR scanner. We thus avoided the need to administer any sedative agent, which was in accordance with our principle of non-use.

### Data Analysis

#### Whole-Brain Analysis

All processing of imaging data was performed using software developed by SUIT, version 3.8 [17, 18, 28], which was executed by the Statistical Parametric Mapping 12 application (SPM12, Wellcome Department of Cognitive Neurology, UK). First, for each imaging data, total cerebellar structures were delineated and segmented into gray and white matter. Each volume, i.e., total intracerebellar volume (TIV), gray matter volume (GMV), or white matter volume (WMV), was estimated using SPM12.

Cerebellar image data were processed using the following multistep procedure: (a) setting origin landmark; (2) transformation and normalization; (3) nonlinear registrations via co-registrations to T_1_ structural brain images of each participant to SUIT T_1_ template space; and (4) a voxel-by-voxel calculation of group statistics on the spatially normalized maps following spatial smoothing with a 3-mm, full-width-at-half-maximum Gaussian kernel.

#### Region of Interest Analysis

When cerebellar analysis detected a difference in lobules, the relative lobular volume ratio was calculated to isolate and confirm the differences between groups. The ratio was defined as: Relative lobular volume ratio = Lobular volume in native space (mm^3^)/Total intracerebellar volume (mm^3^). Multiple lobular volumes were extracted from the native-space images of each participant using scripts from the SUIT toolbox.

#### Multiple Correlation Analysis

In the lobules detected by the region of interest (ROI) analysis, multiple correlation analyses were performed with *z*-transformed values derived from all correlation coefficients on the following points, (a) cerebro-cerebellar volume correlation between whole brain volumetric values and each lobules volumetric values, and (b) cerebellar volume-behavior correlation between each lobules volumetric value and clinical behavioral scores. The scores were derived from HQ, AQ, LOI, VABS_mal, and Kohs_IQ, as well as body mass index (BMI). One item in the HQ, the age of onset of hyperphagia, did not load onto any factor and was deleted from subsequent analyses.

### Statistical Analysis

Whole-brain analysis was performed using a voxel-based comparison between the two groups (PWS and TD controls) using a full factorial design with total intracerebellar volume, age, and sex as covariates to eliminate collinearity with global volume differences and age and sex-related alteration. Category (PWS vs TD) effects were analyzed using a two-tailed *t* test, and a *p* value (*p*) < 0.05 (family-wise error corrected) was considered statistically significant. In the subsequent complementary lobule- and nucleus-specific ROI analysis, two-way repeated measure analysis of variance was utilized, with category and region as covariates and post-hoc pairwise comparisons (Holm’s method) with *p* < 0.01 considered statistically significant. Multiple correlation analysis was performed independently using linear correlation analysis with *p* < 0.05 (false discovery rate corrected). All statistical analyses were performed using SPM12 with MATLAB 2019a (Mathworks, Natick, MA), Sigmaplot 12.5 (SYSTAT Software Inc. San Jose, CA), and R version 3.3.2 (http://www.r-project.org). The graphical presentations were created using R and Python on Anaconda (https://www.anaconda.com/).

## Results

### Participants

Comorbidities in the PWS group were comprised of diabetes, scoliosis, and hypercholesterolemia. Four individuals with PWS were being treated with growth hormone.

### Whole-Cerebellum Analysis

**Fig. 1:**
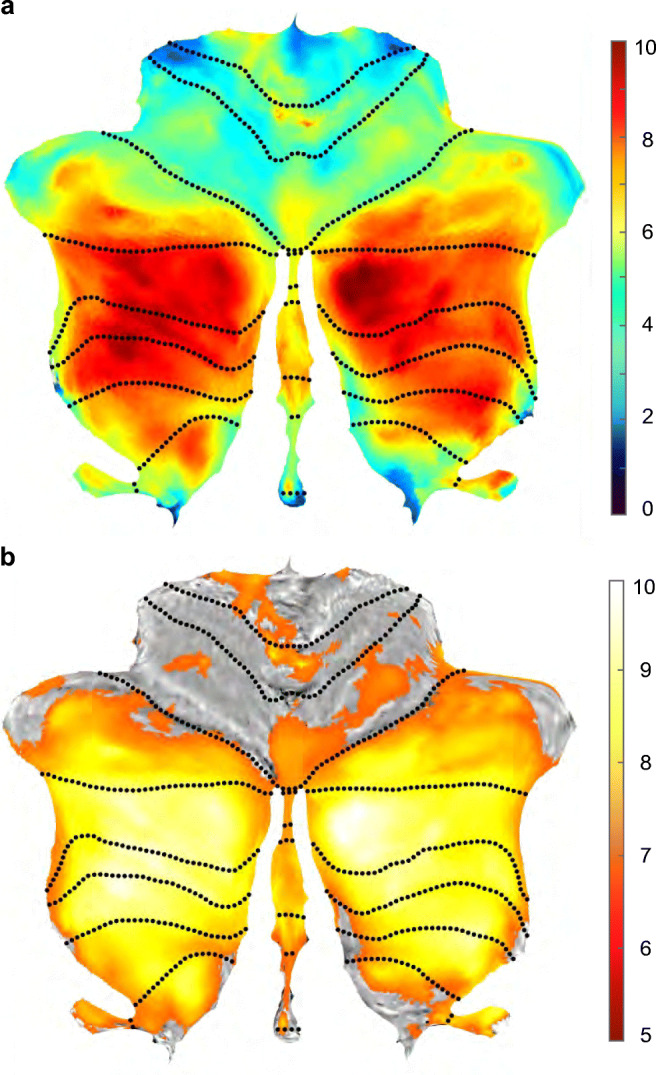
Statistical t-maps of volumetric data overlaid onto the spatially unbiased infratentorial template (SUIT) T1 template space. **a** Differences in the cerebellar volume of gray-matter structures in patients with Prader-Willi syndrome (PWS) compared with healthy controls with typical development (TD). Vertical color bars: *t* values; dotted lines: lobule boundaries. Decreased volumes are observed over lobular subregions Crus I, Crus II, and lobules VIIb, VIIIa, VIIIb, IX, and X. A global volume reduction is also observed. **b** Visualization of significant changes in regional volumes corrected for global volume (= total intracerebellar volume estimated by SUIT). Significance level: *p* < 0.05 (family-wise error corrected)

The results of the voxel-based *t* tests are presented as a flat representation of the cerebellum in Fig. 1. Figure 1a shows that individuals with PWS exhibited global cerebellar volume reductions with significantly decreased relative lobular volumes (TIV; mean [standard deviation], 1014.1 [93.0] mm^3^; *p* < 0.05, family-wise error corrected) compared with those in the TD control group (1191.1 [102.4] mm^3^). The lobule-specific distributions with global scaling using TIV are visualized in Fig. 1b, and statistical data are shown in Table 2. The lobules detected were distributed in the Crus I, Crus II, lobules VIIb, VIIIa, VIIIb, IX, and X bilaterally, and in the vermis VIIIa, VIIIb, and IX. No increases in volume or area relative to controls were detected.

**Table 2:** Statistical data of the cerebellar lobule-specific volume analysis with global scaling using total intracerebellar volume

Set		Cluster				Peak					Coordinate
*p*	c	*p* (FWEc)	*p* (FDRc)	equivk	*p* (unc)	*p* (FWEc)	*p* (FDRc)	*T*	equiv*Z*	*p* (unc)	x,y,z {mm}
0	9	0	5.96e-69	10,833	1.32e-69	0.00014	0.08487	7.2727	6.0768	6.13e-10	13, − 85, − 40
						0.00041	0.08487	6.9455	5.8738	2.13e-09	17, − 77, − 43
						0.00119	0.09261	6.6302	5.6721	7.05e-09	24, − 74, − 56
		0	6.45e-73	11,855	7.16e-74	0.00029	0.08487	7.0519	5.9405	1.42e-09	− 34, − 55, − 56
						0.00045	0.08487	6.9194	5.8574	2.35e-09	− 33, − 68, − 55
						0.00065	0.08486	6.8074	5.7862	3.6e-09	− 40, − 72, − 46
		4.92e-07	2.16e-05	213	9.6e-06	0.00350	0.12532	6.3044	5.4571	2.42e-08	− 1, − 36, − 57
		0.00183	0.045892	33	0.03569	0.00580	0.18901	6.1509	5.3535	4.31e-08	− 48, − 71, − 33
		0.00071	0.02080	48	0.01386	0.01112	0.32467	5.9511	5.2163	9.13e-08	26, − 36, − 46
		3.44e-07	2.01e-05	223	6.71e-06	0.01275	0.34327	5.9087	5.1869	1.07e-07	− 8, − 52, − 57
		5.5e-05	0.00193	97	0.00107	0.01616	0.41939	5.8349	5.1353	8.0e-08	− 15, − 48, − 56
		0.00852	0.187644	13	0.16680	0.02574	0.60981	5.6882	5.0312	2.43e-07	− 45, − 53, − 50
		0.03642	0.72329	1	0.72329	0.04537	0.90521	5.5063	4.9017	4.76e-07	3, − 63, − 4

*c* corrected, *FDR* false discovery rate, *FWE* family wise error, *equivk* equivalent voxel numbers, *T* t-statistics, *unc* uncorrected, *Z z* score, coordinate

### ROI Analysis

The regional relative volume ratios in all lobules and deep gray-matter structures are summarized in Table 3. The same pattern of distribution of significantly decreased relative volume ratios was confirmed. Furthermore, in individuals with PWS, increased relative volume ratios were found in the bilateral cerebellar dentate nuclei (cDN) (mean [standard deviation](× 10^−3^); left, 1.58 [0.26], *p* < 0.01; right, 1.67 [0.30], *p* < 0.01) compared with those of the controls (left, 1.39 [0.13]; right, 1.47 [0.19]).

**Table 3:** Cerebellar volumes in individuals with PWS compared with those in controls

Structure	PWS (*n* = 21)			Control (*n* = 40)		
Whole cerebellum						
TIV	**1014.1 (93.0)**			1191.1 (102.4)		
GM	**621.6 (73.5)**			746.5 (72.9)		
WM	*392.5 (41.6)*			444.7 (57.0)		
Regions (TIV-corrected ratios)						
Lobules	Left	Vermis	Right	Left	Vermis	Right
I-IV	2.90 (0.31)	–	3.37 (0.32)	2.99 (0.15)	-	3.40 (0.16)
V	3.97 (0.38)	–	3.80 (0.33)	3.99 (0.21)	-	3.84 (0.22)
VI	8.57 (0.77)	1.64 (0.19)	7.27 (0.60)	8.71 (0.46)	1.70 (0.14)	7.49 (0.47)
Crus I	**11.43 (1.10)**	0.02 (< 0.01)	**10.77 (1.02)**	12.24 (0.64)	0.01 (< 0.01)	11.71 (0.76)
Crus II	**7.94 (0.96)**	0.35 (0.06)	**7.11 (0.93)**	8.92 (0.57)	0.36 (0.04)	8.22 (0.61)
VIIb	**3.88 (0.52)**	0.15 (0.03)	**3.80 (0.58)**	4.54 (0.35)	0.17 (0.02)	4.49 (0.40)
VIIIa	**3.99 (0.54)**	**0.89 (0.13)**	**3.56 (0.57)**	4.73 (0.40)	1.00 (0.09)	4.22 (0.40)
VIIIb	**3.30 (0.41)**	**0.46 (0.06)**	**3.10 (0.43)**	3.82 (0.36)	0.51 (0.05)	3.58 (0.32)
IX	2.72 (0.27)	*0.61 (0.05)*	*2.90 (0.26)*	2.90 (0.28)	0.65 (0.07)	3.10 (0.28)
X	**0.55 (0.06)**	0.35 (0.07)	**0.51 (0.06)**	0.62 (0.06)	0.35 (0.04)	0.58 (0.05)
Nuclei	Left		Right	Left		Right
Dentate	*1.58 (0.26)*	-	*1.67 (0.30)*	1.39 (0.13)	-	1.47 (0.19)
Interposed	0.21 (0.03)	-	0.20 (0.03)	0.19 (0.03)	-	0.19 (0.03)
Fastigial	0.04 (< 0.01)	-	0.04 (< 0.01)	0.04 (0.01)	-	0.04 (0.01)

Data are represented as mean (standard deviation). Volumes are in mm^3^. *Italic*, significantly different from control at *p* < 0.01; bold, significantly different from control at *p* < 0.0001; GM, gray matter; PWS, Prader-Willi syndrome; controls, typical development; TIV, total intracerebellar volume; TIV-corrected ratio, (regional volume/TIV) × 10^−3^; WM, white matter

### Multiple Correlation Analysis

#### a) Cerebro-cerebellar Volume Correlation Analysis

**Fig. 2:**
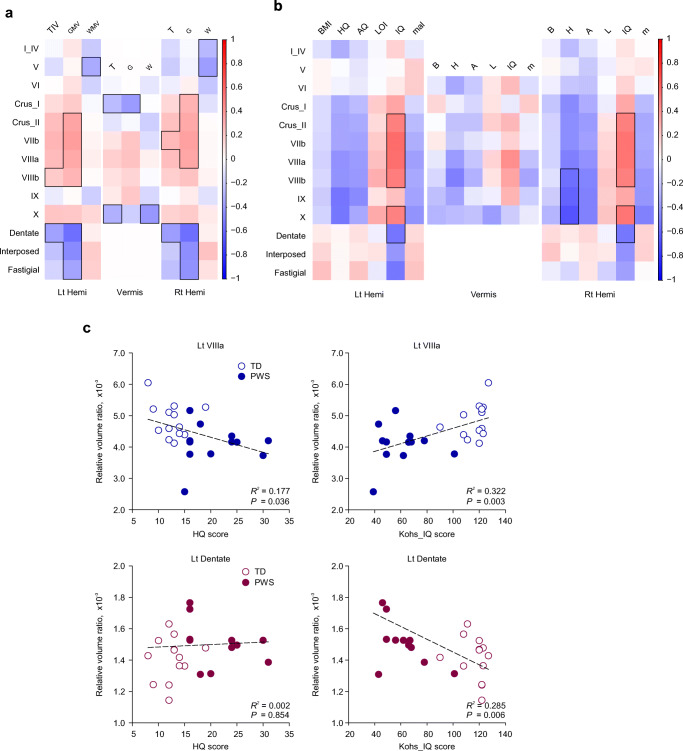
**a** Multiple correlation analysis between regional cerebellar volumes and whole brain volumetric values in individuals with PWS and TD controls. **b** Multiple correlation analysis between regional volumes and clinical behavioral variables in individuals with PWS and TD controls. Color indicates correlation coefficients between the global-volume-corrected volume of a lobule or deep cerebellar nucleus and the behavioral variable indicated above the column. Vertical color bar indicates positive and negative values by red and blue color, respectively. Bolded margins: *p* < 0.05 (error discovery rate corrected). **c** Scatter plots for lobules where significant differences are detected. Horizontal and vertical axes represent the total scores of behavioral assessment scales and global-corrected lobular volumes, respectively. Lt, left; Rt, right; Hemi, hemisphere; BMI (B), body mass index; HQ (H), hyperphagia questionnaire; AQ (A), autism spectrum quotient; LOI, Leyton obsessional inventory; IQ, intelligence quotient from Kohs block test; mal (m), maladaptive behavior score derived from the Vineland adaptive behavior scale-second edition

A heatmap plot that represents the correlation matrix between the regional relative cerebellar volume ratios and the whole brain volumetric values is shown in Fig. 2a. The numerical values of correlation coefficients are summarized in Table S1 and S2 in the electronic supplementary material. Whereas whole brain volume showed a significantly positive correlation only in left VIIIb, right VIIb, and a negative correlation in vermis Crus I, lobule X, and bilateral dentate nuclei, gray matter volume showed bidirectional correlations in a wide range of lobules: positive correlations in right Crus I, bilateral Crus II, VIIb, VIIIa, and left VIIIb; and negative correlations in vermis Crus I, bilateral dentate, interposed, and fastigial nuclei. Finally, white matter volume was negatively correlated with right lobule I_IV, bilateral lobule V, and vermis Crus I.

#### b) Cerebellar Volume-Behavior Correlation Analysis

A heatmap plot that represents the correlation matrix between the regional relative volume ratios and the clinical variables is depicted in Fig. 2b, and the scatterplots of significant regions are shown in Fig. 2c.The numerical values of correlation coefficients are summarized in Table S3 and S4 in the electronic supplementary material. In Fig. 2b, in the areas where significant differences in volume ratio were detected, *z*-transformed heatmap visualization showed bidirectional correlations in a region-dependent manner: (a) HQ, AQ, and VABS_mal were negatively correlated with the volumes of lobules Crus I, Crus II, VIIb, VIIIa, VIIIb, IX, and X, but Kohs_IQ and LOI were positively correlated. (b) In the cDN, however, Kohs_IQ and LOI showed a negative correlation with the volumes.

## Discussion

This study clearly demonstrates that global and local volume differences indeed exist in cerebellar structures in individuals with PWS. While significant relative volume reductions were observed in the posterior inferior lobules, as well as in total intracerebellar volume, increased volume ratios were detected in the cDN. Both results are consistent with functional localization and correlated with clinical behavioral and developmental variables. While the correlation with whole brain volume was not significant except for sparse and scattered lesions, the correlation with gray matter volume was significant. Thus, it is strongly suggested that the reduction in cerebellar volume reflects a distribution corresponding to functional localization, rather than whole brain hypoplasia.

A growing body of evidence has revealed functional topographic representations in the cerebellum, indicating lobule-specific contributions to sensorimotor, cognitive, and higher-order behavioral domains [29, 30, 31]. The lobular structural alterations detected in the present study are highly consistent with the domains that reflect the developmental behavioral characteristics of PWS. The lobules VIIb and VIIIa have been considered to participate in the cognitive domain, which plays an essential part in the developmental delay observed in PWS. Social and behavioral characteristics in PWS may be associated with alterations in the Crus I and II lobules VIIIa and VIIIb, as demonstrated by intrinsic functional connectivity studies [32]. Furthermore, the vermis VIIIa, VIIIb, and IX may account for emotional and homeostatic dysregulations, as these appear to connect to periaqueductal gray matter [33]. From an evolutionary viewpoint, given an emerging finding of triple representations, i.e., anterior lobe, Crus I and II versus Crus I, II, and posterior lobe, versus lobules IX and X, as mirrored by functional organization [34], our results could imply an evolutionary origin of the developmental diaschisis hypothesis, as opposed to spared superior lobules, associated with primary sensorimotor and verbal functions [35].

While the most prominent behavioral characteristic in PWS is hyperphagia, extensive observations have delineated a spectrum of concurrent characteristics such as ASD, obsessive and repetitive behavior, and intellectual disability, all of which predispose an individual to maladaptive conditions. The significant correlation between volume differences and each behavioral variable indicates that cerebellar developmental abnormalities are produced in a lobule-dependent manner. While the relationship between lobule IX and AQ and HQ is consistent with functional topography, the correlation between X and HQ might be due to the cerebellar-hypothalamic pathway. In a sense, such a constellation of involvement detected in the present study may, in part, be explained as a developmental form of the presentation of multi-domain involvements, namely, CCAS [36]. The affective component of CCAS has been associated with the posterior cerebellar lobe, including the midline structures such as the vermis. This has been supported by clinical lesion-symptom observations, including cognitive and emotional disability in children with cerebellar malformation, children surgically treated for brain tumor [37, 38], and a growing evidence of functional connections between limbic and brainstem structures. Considering the affective dysregulation observed in PWS, applying the concept of CCAS to behavioral symptomatology in PWS may lead to an improved understanding and effective interventional strategy for PWS.

In contrast, the dentate volume alteration indicates a developmental abnormality in the major “gateway” structure that receives the efferent pathway from the cerebellar peduncle, inferior olive, and pons, and the afferent projection toward extracerebellar structures. The cDN has also been considered not only a motor domain but also a cognitive domain, as revealed by neuroanatomical and high spatio-temporal resolution resting-state fMRI analyses that identified three distinct functional territories within the cDN that contribute uniquely to default-mode, salience-motor, and visual cerebral cortical networks [39]. Furthermore, an unfolded map of the cDN based on recent microstructural and neuroimaging investigations has revealed spatially segregated motor and non-motor domains localized in the dorsal and ventral portions of cDN, respectively [39, 40]. The cerebellar-hypothalamic pathway has also been highlighted [41]. Both animal and human studies have uncovered a reciprocal circuit via the middle and posterior cerebellar lobules and crus, which highly implicates a potential contribution to hyperphagic behavior including visceral and homeostatic dysregulation in PWS [42, 43]. Given the functional correlation that has also been clinically observed, such as the cDN and cognitive function in patients with involvement of cDN [44], our current findings strongly suggest that the deep cerebellar nuclei including the cDN also contribute to the developmental behavioral characteristics in PWS.

Although systematic neuropathological analysis of the cerebellum in PWS remains sparse, a number of autopsy reports have documented the reduced number and size of Purkinje cells and an increased gliotic reaction, which indicate developmental abnormalities [45, 46, 47], as disorder-specific cerebellar microstructural evidence has been reported in a variety of neurodevelopmental disorders (e.g., ASD) [48]. These reports have also depicted a characteristic dentate structure, namely, relative undulating structures and grumose degeneration. Considering the maturational process of the cDN rather as a volume reduction together with extensive folding transformation without any other neurodegenerative features [49], our current findings encourage us to hypothesize that arrested maturation in the cDN exists in the brain in individual with PWS, as detected quantitatively by MRI. Further investigations could provide better neuropathological-neuroimaging correlates in specific areas that are vital to the behavioral maturation in PWS. More focused MR microscopy and advanced neuroimaging based on ultra-high field systems are highly warranted to reveal the dorsal-to-ventral gradient of undulating structures in the cDN and functional connection with non-motor cortical areas reflecting outputs affected by PWS (e.g., by diffusion imaging, susceptibility-weighted imaging, and T_1_-fluid attenuated inversion recovery imaging).

Whether the volume alterations are a result of genetics or the environment remains controversial. Previous reports have shown that individuals with morbid obesity also exhibit a similar reduction in cerebellar volume, although not as prominently as in PWS [50]. A significant negative correlation between the volume of cerebral cortex and BMI and the volume recovery after metabolic surgery has been demonstrated in obese adults [51]. In contrast, the divergent findings in monozygotic twins and the lack of correlation with BMI in childhood indicate functional representation (e.g., executive function and aging) [52]. Although there may be differences in developmental volume trajectories between cerebral and cerebellar cortices [53], epigenetic changes may be added to the observable alterations based on genetically constrained differences. Given the nutritional phase and diagnostic timing-dependent obesity typically observed in PWS [54], longitudinal analysis from infancy to adolescence of body-weight trajectories may elucidate both genetic and epigenetic contributions during the life of individuals with PWS, leading to rational and efficient treatment strategies based on biological evidence [55].

From a technical neuroimaging standpoint, interpretations should be made cautiously; magnetic susceptibility effects, which cause non-linear distortions of images, are likely to be pronounced at the posterior inferior part of the brain, as possible flow artifacts and head motion might substantially influence the image quality despite the use of flow compensation and well-organized, pre-scan preparation techniques, respectively. Moreover, the small number of participants included in the paired subgroup analysis is a limitation of this study. The rarity of PWS syndrome (e.g., an overall incidence of one in 16,000 births in Western Japan) [56] is a main factor that affected our ability to recruit more participants in a limited time frame. Furthermore, the CCAS/Schmahmann syndrome scale was not utilized for neuropsychological assessment because the possible inaccuracy leading to false conclusion should be excluded when applying the scale to the children and participants with developmental delay. Further analyses with a larger number of participants balanced for genetic subtype along with assessment using the CCAS/Schmahmann scale are highly warranted for better understanding of the cerebro-cerebellar crosstalk based on genetic and self-organizing processes in the neurodevelopmental pathophysiology of PWS.

## Conclusion

The present study provides an objective evidence of lobular-specific developmental abnormalities of the cerebellum in individuals with PWS. The altered lobular and deep cerebellar nuclei volumes indicate attendant developmental abnormalities within the cerebellar structures, which are associated with commonly observed clinical and behavioral patterns in PWS. Therefore, the significant contribution of altered functional connectivity implied by our results should encourage research focusing on the cerebellum as a crucial structure underlying the pathophysiology of the brain in individuals with PWS.

## Electronic Supplementary Material

ESM 1 (DOCX 38 kb)
