# Supplementary material for: Cerebellar Volumes Associate with Behavioral Phenotypes in Prader-Willi Syndrome
Source: Cerebellum. 2020 Jul 13;19(6):778–87. doi: 10.1007/s12311-020-01163-1 (PMC7588377; doi:10.1007/s12311-020-01163-1)
Supplement: Supplementary file 1 — (DOCX 38 kb) [file 12311_2020_1163_MOESM1_ESM.docx]

| Hemisphere |  | BMI | HQ | AQ | LOI | Kohs_IQ | V_mal |
| --- | --- | --- | --- | --- | --- | --- | --- |
| I_IV | L | -0.112 | -0.306 | -0.160 | -0.021 | 0.174 | -0.086 |
|  | R | -0.054 | -0.251 | -0.111 | -0.015 | 0.109 | 0.002 |
| V | L | 0.039 | -0.004 | -0.092 | -0.011 | 0.019 | 0.197 |
|  | R | 0.022 | -0.071 | -0.203 | -0.053 | 0.066 | 0.098 |
| VI | L | 0.037 | -0.049 | -0.076 | -0.008 | 0.089 | 0.193 |
|  | R | -0.056 | -0.213 | -0.236 | -0.001 | 0.166 | -0.038 |
| Crus_I | L | -0.134 | -0.327 | -0.259 | 0.205 | 0.342 | -0.154 |
|  | R | -0.229 | -0.448 | -0.314 | -0.064 | 0.337 | -0.263 |
| Crus_II | L | -0.137 | -0.343 | -0.329 | 0.131 | 0.500* | -0.263 |
|  | R | -0.173 | -0.431 | -0.383 | 0.104 | 0.492* | -0.336 |
| VIIb | L | -0.145 | -0.345 | -0.350 | 0.203 | 0.578** | -0.304 |
|  | R | -0.166 | -0.434 | -0.368 | 0.145 | 0.549** | -0.320 |
| VIIIa | L | -0.173 | -0.420 | -0.389 | 0.244 | 0.568** | -0.370 |
|  | R | -0.169 | -0.492 | -0.424 | 0.175 | 0.549** | -0.350 |
| VIIIb | L | -0.142 | -0.411 | -0.395 | 0.308 | 0.500* | -0.332 |
|  | R | -0.117 | -0.562** | -0.475 | 0.211 | 0.496* | -0.395 |
| IX | L | -0.195 | -0.532 | -0.442 | 0.110 | 0.238 | -0.355 |
|  | R | -0.195 | -0.601** | -0.485 | 0.195 | 0.334 | -0.403 |
| X | L | -0.162 | -0.520 | -0.335 | 0.326 | 0.486* | -0.420 |
|  | R | -0.286 | -0.661** | -0.488 | 0.224 | 0.532** | -0.486 |
| Dentate | L | 0.079 | 0.039 | 0.117 | -0.056 | -0.534** | 0.132 |
|  | R | 0.131 | 0.084 | 0.143 | -0.047 | -0.527** | 0.164 |
| Interposed | L | 0.151 | 0.026 | 0.086 | 0.105 | -0.401 | 0.201 |
|  | R | 0.218 | 0.030 | 0.106 | 0.080 | -0.427 | 0.174 |
| Fastigial | L | 0.234 | 0.029 | 0.225 | -0.079 | -0.525** | 0.229 |
|  | R | -0.062 | -0.148 | 0.097 | -0.180 | -0.366 | 0.013 |

Table S1. Correlation coefficients matrix between the lobule volumes in cerebellar hemisphere and the behavioral characteristic scores

** indicates *p* < 0.01 (2-tailed) and * indicates *p* < 0.05 (2-tailed) (false discovery error corrected).

BMI, body mass index; HQ, hyperphagia questionnaire; AQ, autism spectrum quotient; LOI, Leyton obsessional inventory; Kohs_IQ, intelligence quotient from Kohs block test; V_mal, maladaptive behavior score derived from the Vineland adaptive behavior scale-second edition.

Table S2. Correlation coefficients matrix between the lobular volumes in cerebellar vermis and the behavioral characteristic scores

| Vermis |  | BMI | HQ | AQ | LOI | Kohs_IQ | V_mal |
| --- | --- | --- | --- | --- | --- | --- | --- |
| VI |  | -0.087 | -0.336 | -0.204 | 0.096 | 0.253 | -0.129 |
| Crus_I |  | 0.150 | -0.063 | -0.159 | 0.105 | -0.074 | 0.208 |
| Crus_II |  | -0.007 | -0.230 | -0.237 | 0.190 | 0.273 | -0.063 |
| VIIb |  | 0.077 | -0.202 | -0.105 | -0.059 | 0.170 | -0.058 |
| VIIIa |  | -0.081 | -0.386 | -0.219 | 0.166 | 0.440 | -0.238 |
| VIIIb |  | -0.148 | -0.497 | -0.295 | 0.107 | 0.356 | -0.341 |
| IX |  | -0.244 | -0.359 | -0.089 | -0.051 | 0.289 | -0.185 |
| X |  | -0.280 | -0.251 | -0.232 | -0.372 | -0.198 | -0.078 |

** indicates *p* < 0.01 (2-tailed) and * indicates *p* < 0.05 (2-tailed) (false discovery error corrected).

BMI, body mass index; HQ, hyperphagia questionnaire; AQ, autism spectrum quotient; LOI, Leyton obsessional inventory; Kohs_IQ, intelligence quotient from Kohs block test; V_mal, maladaptive behavior score derived from the Vineland adaptive behavior scale-second edition.

Table S3. Correlation coefficients matrix between lobule volumes in the cerebellar hemisphere and behavioral characteristic scores

| Hemisphere |  | TIV | GMV | WMV |
| --- | --- | --- | --- | --- |
| I_IV | L | -0.029 | 0.091 | -0.209 |
|  | R | -0.144 | -0.029 | -0.280* |
| V | L | -0.121 | 0.035 | -0.328* |
|  | R | -0.199 | -0.044 | -0.379** |
| VI | L | 0.008 | 0.119 | -0.171 |
|  | R | -0.060 | 0.059 | -0.230 |
| Crus_I | L | 0.134 | 0.208 | -0.028 |
|  | R | 0.158 | 0.295* | -0.111 |
| Crus_II | L | 0.255 | 0.354** | 0.011 |
|  | R | 0.241 | 0.333* | 0.014 |
| VIIb | L | 0.282* | 0.372** | 0.045 |
|  | R | 0.271* | 0.370** | 0.026 |
| VIIIa | L | 0.263 | 0.334* | 0.062 |
|  | R | 0.250 | 0.343** | 0.020 |
| VIIIb | L | 0.205 | 0.283* | 0.015 |
|  | R | 0.186 | 0.239 | 0.040 |
| IX | L | -0.072 | 0.012 | -0.181 |
|  | R | <0.001 | 0.071 | -0.113 |
| X | L | 0.223 | 0.220 | 0.153 |
|  | R | 0.180 | 0.202 | 0.084 |
| Dentate | L | -0.367** | -0.566** | 0.069 |
|  | R | -0.372** | -0.565** | 0.057 |
| Interposed | L | -0.234 | -0.453** | 0.190 |
|  | R | -0.202 | -0.432** | 0.230 |
| Fastigial | L | -0.168 | -0.353** | 0.181 |
|  | R | -0.247 | -0.426** | 0.118 |

** *p* < 0.01 (2-tailed) and * *p* < 0.05 (2-tailed; false discovery error corrected).

GMV, gray matter volume; TIV, total intracranial volume; WMV, white matter volume.

Table S4. Correlation coefficients matrix between lobular volumes in the cerebellar vermis and behavioral characteristic scores

| Vermis |  | TIV | GMV | WMV |
| --- | --- | --- | --- | --- |
| VI |  | -0.036 | 0.010 | -0.097 |
| Crus_I |  | -0.269* | -0.381** | -0.002 |
| Crus_II |  | -0.103 | -0.030 | -0.186 |
| VIIb |  | 0.101 | 0.222 | -0.125 |
| VIIIa |  | 0.186 | 0.232 | 0.051 |
| VIIIb |  | 0.111 | 0.165 | -0.013 |
| IX |  | 0.086 | 0.218 | -0.150 |
| X |  | -0.309* | -0.186 | -0.401** |

** *p* < 0.01 (2-tailed) and * *p* < 0.05 (2-tailed; false discovery error corrected).

GMV, gray matter volume; TIV, total intracranial volume; WMV, white matter volume.
